# miR-101 suppresses the epithelial-to-mesenchymal transition by targeting *ZEB1* and *ZEB2* in ovarian carcinoma

**DOI:** 10.3892/or.2014.3106

**Published:** 2014-03-21

**Authors:** FEI GUO, DAVID COGDELL, LIMEI HU, DA YANG, ANIL K. SOOD, FENGXIA XUE, WEI ZHANG

**Affiliations:** 1 Department of Pathology, The University of Texas M.D. Anderson Cancer Center, Houston, TX 77030, USA; 2 Department of Gynecologic Oncology and Reproductive Medicine, The University of Texas M.D. Anderson Cancer Center, Houston, TX 77030, USA; 3 Center for RNAi and Non-Coding RNA, The University of Texas M.D. Anderson Cancer Center, Houston, TX 77030, USA; 4 Department of Gynecology and Obstetrics, Tianjin Medical University General Hospital, Tianjin 300052, P.R. China

**Keywords:** ovarian carcinoma, epithelial-to-mesenchymal transition, miR-101, ZEB1, ZEB2

## Abstract

Ovarian carcinoma is the most lethal gynecologic malignancy; the majority of patients succumb to the disease within 5 years of diagnosis. The poor survival rate is attributed to diagnosis at advanced stage, when the tumor has metastasized. The epithelial-to-mesenchymal transition (EMT) is a necessary step toward metastatic tumor progression. Through integrated computational analysis, we recently identified a master microRNA (miRNA) network that includes miR-101 and regulates EMT in ovarian carcinoma. In the present study, we characterized the functions of miR-101. Using reporter gene assays, we demonstrated that miR-101 suppressed the expression of the E-cadherin repressors ZEB1 and ZEB2 by directly targeting the 3′-untranslated region (3′UTR) of both ZEB1 and ZEB2. Introduction of miR-101 significantly inhibited EMT and cell migration and invasion. Introducing cDNAs of *ZEB1* and *ZEB2* without 3′UTR abrogated miR-101-induced EMT alteration, respectively. Our findings showed that miR-101 represents a redundant mechanism for the miR-200 family that regulates EMT through two major E-cadherin transcriptional repressors.

## Introduction

Ovarian carcinoma is the most lethal gynecologic malignancy in the United States (1). Approximately 23% of gynecologic cancers originate in the ovary, but 47% of all deaths from cancer of the female genital tract occur in women with ovarian cancer (2). The high rate of lethality from ovarian carcinoma is mainly due to the advanced stage (stage III and IV) of disease at the time of diagnosis and lack of effective therapies for advanced disease (2). A better understanding of molecular changes in ovarian carcinoma is required to identify better therapeutic strategies for this fatal disease.

The epithelial-to-mesenchymal transition (EMT) has been recognized as an important physiological process that is associated with cancer progression and metastasis in multiple epithelial cancer types, including ovarian carcinoma (3). A hallmark of EMT is the loss of epithelial markers (such as E-cadherin) and gain of mesenchymal markers (such as vimentin, N-cadherin). The change of cellular lineage has been extensively studied and we have begun to appreciate the signaling pathways involved. Signaling molecules that affect EMT of ovarian carcinoma include MAPK (4), Notch3 (5), SFRP4 (6) and ALX1 (7). A major area for EMT investigations has been the key cell membrane protein E-cadherin, which is responsible for holding neighboring epithelial cells together in a classic cobblestone structure. During EMT, E-cadherin is invariably lost from the membrane, allowing cells to assume a loosely connected structure that is associated with enhanced cell migration/invasion, thus contributing to metastasis. A number of transcription factors have been shown to be key regulators of E-cadherin expression, including SNAI1, SNAI2, ZEB1 and ZEB2.

The regulatory circuit of E-cadherin has been further illustrated by the discoveries that these key transcriptional factors are post-transcriptionally regulated by a group of microRNAs (miRNAs) (8–14). miRNAs are short 20–22-nucleotide RNA molecules that are negative regulators of gene expression in a variety of eukaryotic organisms. Single-stranded miRNAs bind to specific target mRNAs through partly complementary sequences that are predominantly in the 3′-untranslated region (3′-UTR) (15,16). The best-characterized miRNAs that suppress EMT belong to the miR-200 family (miR-200a, miR-200b, miR-200c, miR-141, miR-429), which have been shown to directly target the 3′-UTRs of *ZEB1* and *ZEB2* (13,17). Recent studies identified miR-506 as one of a new class of robust EMT repressors; miR-506 directly targets the 3′-UTRs of *SNAI2* (18,19), *CD151* (19) and vimentin (*VIM)* (19). Our investigations of the miRNA network regulating the EMT of ovarian carcinoma have identified miR-101 as another key regulator (18). miR-101 has been shown by recent studies to regulate EMT through EZH2 (20,21) and the Wnt signaling pathway (22). However, how miR-101 suppresses EMT in ovarian carcinoma has not been reported; this is the focus of the investigation reported here.

In the present study, we comprehensively examined the mechanisms through which miR-101 regulates EMT in ovarian carcinoma. We provide evidence that miR-101 directly targets ZEB1 and ZEB2 through an miR-200-independent mechanism.

## Materials and methods

### Materials and cell culture

The ovarian cancer cell line SKOV3 was obtained from American Type Culture Collection (Manassas, VA, USA) and maintained in RPMI-1640 medium supplemented with 10% fetal bovine serum (FBS). The cells were incubated at 37°C in an atmosphere containing 5% CO_2_. Human miR-101 mimics hsa-miR-101 and hsa-miR-Ctrl were obtained from Dharmacon (Chicago, IL, USA). Small-interfering RNA (siRNA) targeting *ZEB1* or *ZEB2* and scrambled negative siRNA control were purchased from Sigma (St. Louis, MO, USA).

### miRNA/siRNA transfection

Cells (2×10^5^/well) were seeded in 6-well plates and allowed to attach for at least 16 h. miR-101 mimic (miR-101), scrambled negative miR-101 control (miR-Ctrl), or siRNA was transfected into cells using Lipofectamine RNAiMAX (Invitrogen, Grand Island, NY, USA) at a final concentration of 50 nM.

### Luciferase reporter assay

The 3′-UTRs of the *ZEB1* and *ZEB2* genes, each of which contains one or two putative miR-101-binding sites, were amplified by polymerase chain reaction (PCR) from cDNA derived from HeyA8 cells and inserted into the multiple cloning site of the pmirGLO vector (Promega, Fitchburg, WI, USA). The primers used for the *ZEB1* gene 3′-UTR were: 5′-AAACTCGAGTACTTCAATTC CTCGGTATTG-3′, and 5′-AAATCTAGACACACTGTTCTA CAGTCCAAGGC-3′; the primers used for the *ZEB2* gene 3′-UTR were: 5′-AAACTCGAGTACACCCATGTC AGTATTAGAAG-3′, and 5′-AAATCTAGACACAGATCAA CGTCATGTTCC-3′. Two mutant *ZEB1* and *ZEB2* 3′-UTR reporter vectors that lacked the binding sites for miR-101 were created through site-directed mutagenesis using a QuikChange kit (Stratagene, La Jolla, CA, USA). The primers used for site-directed mutagenesis of the *ZEB1* gene 3′-UTR were: *ZEB1* 3′-UTR-M1, 5′-CTGTGCAACATTTTTTGTA CAAATGTCTTCAAACCTGG-3′, and 5′-CCAGGTTTGAA GACATTTGTACAAAAAATGTTGCACAG-3′; *ZEB1* 3′-UTR-M2, 5′-CACAGTGTAGTGTATAAGTGCACAGTTT GTATTAATACAATAATAT-3′, and 5′-ATATTATTGTATTA ATACAAACTGTGCACTTATACACTACACTGT G-3′. The primers used for site-directed mutagenesis of the *ZEB2* gene 3′-UTR were as follows: *ZEB2* 3′-UTR-M, 5′-CCTAATTTTA TTTATTTCAGAGCTCAGTGTACAGTATTATAGTTCTT C-3′, and 5′-GAAGAACTATAATACTGTACACTGAGCTCT GAAATAAATAAAATTAGG-3′. All clones were verified by DNA sequencing.

For the luciferase reporter assay, 0.5 μg of pmirGLO, pmirGLO-3′-UTR-WT, or pmirGLO-3′-UTR-MT was transfected into HeLa cells that were cultured in 24-well plates, together with 50 nM miR-101 or miR-Ctrl, using Lipofectamine 2000 (Invitrogen). Twenty-four hours after transfection, cells were subjected to lysis and firefly luciferase and *Renilla* luciferase activities were determined using a dual-luciferase reporter assay system (Promega) as previously described (23). Relative firefly luciferase activity (firefly luciferase activity/*Renilla* luciferase activity) for each construct was compared to that of the control mimics. For each transfection, luciferase activity was averaged from triplicates.

### Real-time reverse transcription PCR analysis (RT-PCR)

RT-PCR was performed as previously described (18). In brief, total RNA was isolated with the mirVana miRNA isolation kit (Ambion, Grand Island, NY, USA). Reverse transcription was performed using SuperScript II reverse transcriptase (Invitrogen) according to the manufacturer’s protocol. TaqMan real-time PCR assays for *ZEB1* and *ZEB2* were purchased from Applied Biosciences (Grand Island, NY, USA). RNU6B was used as a normalization control.

### Western blot analysis

Primary antibodies for β-actin, fibronectin and vimentin were obtained from Santa Cruz Biotechnology, Inc. (Santa Cruz, CA, USA). The N-cadherin antibody was obtained from Invitrogen [N-Cadherin Monoclonal Antibody, Mouse (3B9)]. The E-cadherin antibody was purchased from BD Biosciences (San Jose, CA, USA). Western blotting was performed as previously described (24). In brief, equal amounts of protein from whole cell lysates of each sample were loaded on a 10% polyacrylamide gel for electrophoresis; the membrane was blocked in 5% non-fat milk in 1X Tris-buffered saline (pH 7.4) containing 0.05% Tween-20 and probed with primary antibodies at concentrations of 1:500 (for E-cadherin), 1:1,000 (for β-actin, fibronectin, N-cadherin), or 1:2,500 (for vimentin). The secondary antibodies were used at a concentration of 1:4,000 to 1:5,000. The proteins were visualized using the SuperSignal West Pico or SuperSignal Femto chemiluminescent substrate from Pierce Biotechnology, Inc. (Rockford, IL, USA).

### Immunofluorescence staining

Immunofluorescence staining was performed as previously described (18). In brief, SKOV3 cells were seeded onto an uncoated glass slide coverslip and cultured in complete medium under standard cell culture conditions. Cells were fixed in 4% paraformaldehyde at ambient temperature for 15 min, followed by permeabilization in 1X phosphate-buffered saline solution (PBS) containing 0.05% NP-40 for 30 min at ambient temperature. The cells were blocked in blocking solution (1X PBS containing 10% normal goat serum and 0.05% NP-40) for at least 4 h. After washing briefly with 1X PBS, the cells were incubated with a mouse monoclonal anti-human E-cadherin antibody (1:100 dilution in the blocking solution) at 4°C overnight. After washing, a goat anti-mouse IgG conjugated with Alexa Fluor 488 (Invitrogen, #A11029; 1:1,000 in the blocking solution) was incubated with the cells at ambient temperature for 1 h. Phalloidin staining was performed at ambient temperature for 45 min using phalloidin-TRITC (Molecular Probes, Invitrogen) at a concentration of 2.5 μg/ml. Phase images were captured by a ZEISS Axiovert 200 microscope at a magnification of ×200. The fluorescence images were captured using a ZEISS Axioplan 2 imaging microscope at a magnification of ×630 or ×400.

### Cell invasion assays

Cell invasion assays were performed in triplicate using Matrigel-coated Transwell chambers (8-μm pore size; BD Pharmingen, Franklin Lakes, NJ, USA). Briefly, cells (5×10^4^) transfected with miR-101 or miR-Ctrl were plated 48 h after transfection in 500 μl of serum-free medium in the upper chamber of the wells and allowed to migrate toward 10% FBS medium (750 μl) in the lower chamber for 22 h. Cells that remained on top of the filter were mechanically removed, and those that migrated to the underside of the filter were fixed and stained with Hema-Diff Solution (Fisher Scientific, Pittsburgh, PA, USA). Invaded cells were counted under a microscope in six randomly chosen fields and representative images were captured. Data are expressed as number of invaded cells (means ± standard deviation) normalized to the number of control cells that migrated. Each result represents an average of triplicates.

### Wound healing assay

SKOV3 cells (~2×10^5^) were plated in each well of a 6-well plate. Following overnight incubation, the cells were transfected with 50 nM miR-Ctrl or 50 nM of miR-101 for 48 h, to allow the cells reached full confluence. The cell monolayers were wounded by scraping with a micropipette tip, washed several times with medium to remove dislodged cells and placed back in growth medium. Images were captured using a phase-contrast microscope (Olympus, Japan) immediately and 16 h after wounding.

### Generation of SKOV3-ZEB1/ZEB2 stable cell lines

The pcDNA3.1(+)-*ZEB1* and pcDNA3.1(+)-*ZEB2* vectors were generated by digesting a *ZEB1* fragment from MGC human *ZEB1* cDNA (MHS4426-211690344) or *ZEB2* from MGC human *ZEB2* cDNA (MHS4426-211690995) (both from Thermo Scientific, Waltham, MA, USA) and subcloning it into the pcDNA3.1(+) vector using *Bam*HI and *Not*I restriction enzymes and *Kpn*I and *Xba*I restriction enzymes, respectively. The correct sequence and orientation were confirmed by DNA sequencing. The pcDNA3.1-ZEB1 or pcDNA3.1-ZEB2 vector or empty vector alone was transfected into SKOV3 cells using Lipofectamine 2000. At 48 h after transfection, the cells were placed in culture in complete medium with 1,000 μg/ml G418 for 4 weeks.

## Results

### miR-101 directly targets ZEB1 and ZEB2

**Figure 1:**
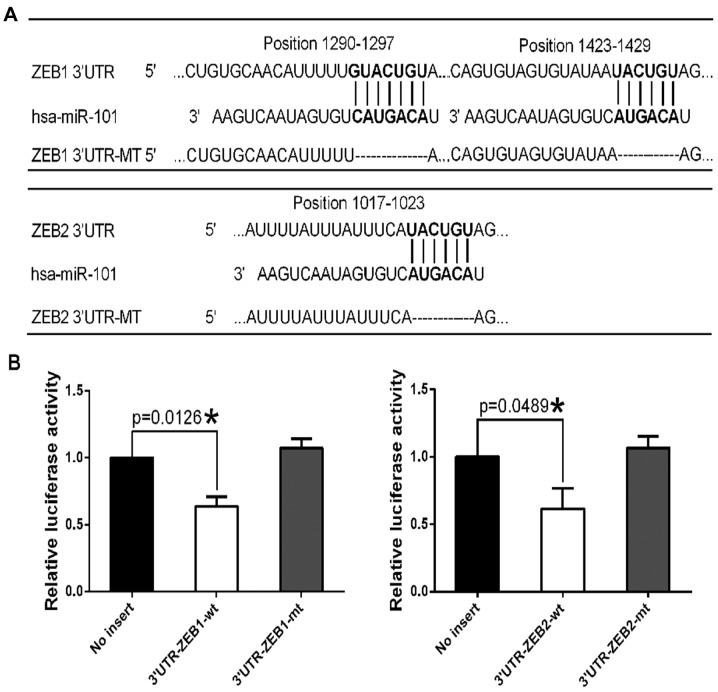
miR-101 directly targets *ZEB1* and *ZEB2*. (A) The two predicted miR-101-binding sites in the *ZEB1* 3′-UTR region are shown in the upper panel and the one predicted miR-101-binding site in the *ZEB2* 3′-UTR region is shown in the lower panel. (B) The pmirGLO-*ZEB1* and pmirGLO-*ZEB2* reporter genes had putative miR-101-binding sites cloned into the pmirGLO-control vector. The pmirGLO-*ZEB1*-mt vector had the two miR-101-binding sites deleted; the deletion was confirmed by sequencing. The pmirGLO-*ZEB2*-mt vector had the one miR-101-binding site deleted, and again the deletion was confirmed by sequencing. HeLa cells were transfected with pmirGLO-*ZEB1* or pmirGLO-*ZEB1*-mt or with pmirGLO-*ZEB2* or pmirGLO-*ZEB2*-mt, together with an miR-101 mimic or mimic negative control. The mean relative luciferase activities are shown, with standard errors, from 3 independent experiments.

TargetScan analysis predicted two miR-101-binding sites in the 3′-UTR of the *ZEB1* gene and one site in the 3′-UTR of the *ZEB2* gene (Fig. 1A), suggesting that miR-101 may directly target *ZEB1* and *ZEB2*. We performed luciferase reporter assays to examine whether miR-101 mimic (miR-101) directly targets *ZEB1* and *ZEB2*. We cloned the 3′-UTR of *ZEB1* or the 3′-UTR of *ZEB2* into the pmirGLO-ctrl vector to generate pmirGLO-ZEB1 and pmirGLO-ZEB2 constructs. Co-transfection of pmirGLO-ZEB1 and miR-101 into HeLa cells resulted in 36.3% less luciferase activity than co-transfection with miR-Ctrl, suggesting that miR-101 directly targets *ZEB1* (Fig. 1B). Similarly, co-transfection of pmirGLO-ZEB2 and miR-101 resulted in 38.6% less luciferase activity than co-transfection with miR-Ctrl, suggesting that miR-101 directly targets *ZEB2* (Fig. 1B).

To confirm that miR-101 specifically regulates *ZEB1* and *ZEB2* through the predicted binding sites, we generated the constructs pmirGLO-ZEB1-mt and pmirGLO-ZEB2-mt, from which the miR-101-binding site sequences on the 3′-UTR of ZEB1 or ZEB2 were deleted. We then co-transfected the mutant constructs with miR-101 or miR-Ctrl into HeLa cells. Deletion of the miR-101-binding sites from the 3′-UTR of *ZEB1* or ZEB2 abolished the effect of miR-101 on luciferase activity (Fig. 1B). These results indicate that the *ZEB1* and *ZEB2* genes are direct targets of miR-101.

**Figure 2:**
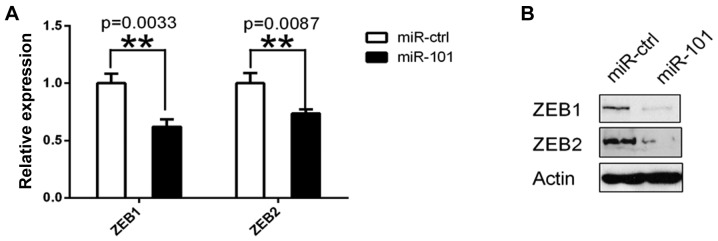
miR-101 suppresses endogenous *ZEB1* and *ZEB2* expression. (A) *ZEB1* and *ZEB2* in SKOV3 cells were quantified by TaqMan real-time PCR. All data are means of triplicate PCR assays. (B) Levels of ZEB1 and ZEB2 proteins in SKOV3 cells transfected with miR-101 mimic (miR-101) or control (miR-Ctrl) were measured by western blotting using whole cell lysates from each sample.

To demonstrate that miR-101 is an endogenous regulator of *ZEB1* and *ZEB2* in ovarian carcinoma cells, we transfected SKOV3 ovarian cancer cells with miR-101 or miR-Ctrl followed by measurement of *ZEB1* and *ZEB2* mRNA and protein at 48 h after transfection. The results showed that *ZEB1* and *ZEB2* mRNA and proteins in SKOV3 cells were significantly downregulated after miR-101 transfection (Fig. 2).

### miR-101 inhibits EMT, migration and invasion of ovarian cancer cells

**Figure 3:**
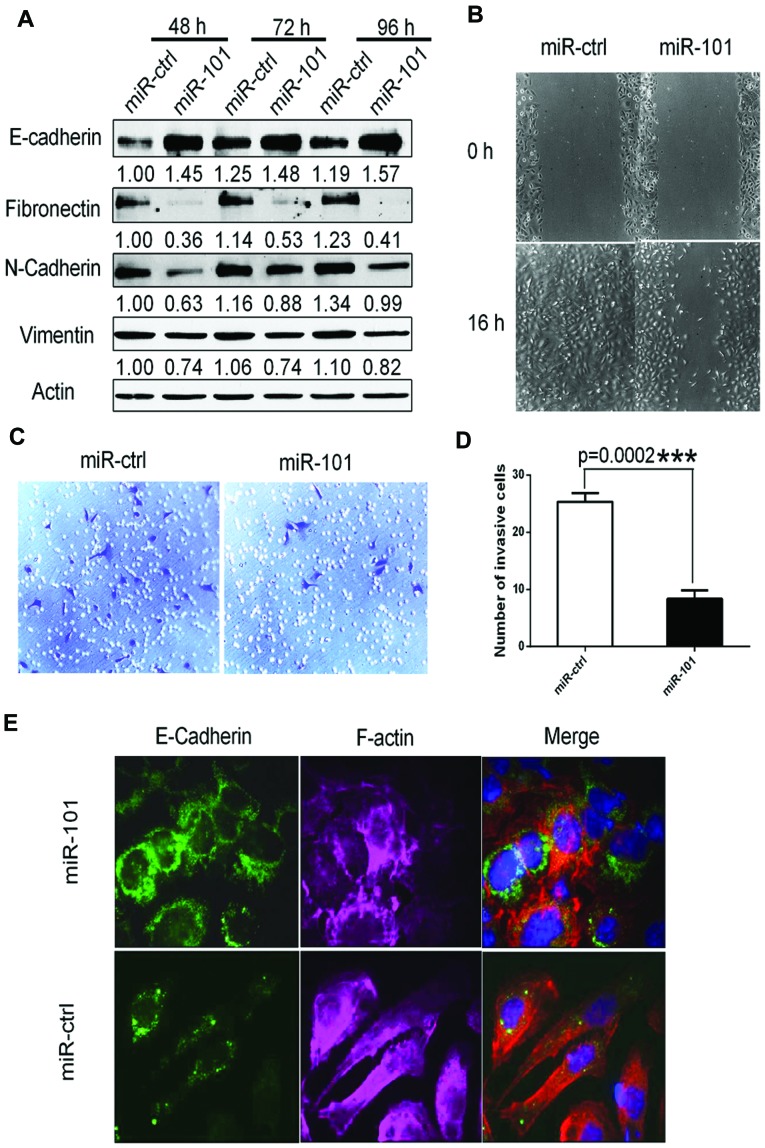
miR-101 suppresses EMT and cell migration and invasion. (A) Levels of E-cadherin, fibronectin, N-cadherin and vimentin proteins in SKOV3 cells after transfection with miR-101 mimic (miR-101) or control (miR-Ctrl) were measured by western blotting using whole cell lysates from each sample. Actin was used to control protein loading. (B) Wound-healing assay was performed in SKOV3 cells. The extent of closure of the wound, representing cell migration, was monitored under phase-contrast microscopy and images were captured at 0 and 16 h. (C and D) Cell invasion assays in SKOV3 cells transfected with miR-101 or miR-Ctrl after 48 h. The cells were allowed to invade toward the lower chamber for 22 h. Each result in (D) represents an average of triplicates. (E) E-cadherin and F-actin immunofluorescence staining of SKOV3 cells transfected with miR-101 or miR101-Ctrl for 72 h. EMT, epithelial-to-mesenchymal transition.

To determine whether forced expression of miR-101 promotes the epithelial phenotype, we transfected SKOV3 cells with either miR-101 or miR-Ctrl. miR-101 overexpression significantly increased E-cadherin protein levels, while it downregulated mesenchymal markers fibronectin, N-cadherin and vimentin (Fig. 3A).

It is well recognized that the EMT is involved in cell migratory and invasive capacity in ovarian carcinoma (6,25). To this end, we performed a wound-healing assay and found that cell migration was markedly reduced in cells expressing ectopic miR-101 when compared with cells transfected with miR-Ctrl (Fig. 3B). Similarly, invasion assays revealed that ectopic miR-101 expression significantly decreased cell invasion by ~3-fold when compared with miR-Ctrl-transfected cells (Fig. 3C and D). We also performed immunofluorescence staining to directly visualize the effect of miR-101 on E-cadherin expression and localization and cell morphology (Fig. 3E). In this experiment, the E-cadherin protein was localized on the membrane at cell-cell junctions of miR-101-transfected SKOV3 cells, which is indicative of epithelial cells. In addition, F-actin distribution was rearranged to a cortical pattern, which is another hallmark of the epithelial phenotype (Fig. 3E). In contrast, the cells transfected with miR-Ctrl showed the elongated mesenchymal cell phenotype indicated by an absence of E-cadherin on the cell membrane and rearrangement of F-actin from a cortical to a stress-fiber pattern (Fig. 3E).

### Inhibition of EMT by miR-101 is mediated by ZEB1 and ZEB2

**Figure 4:**
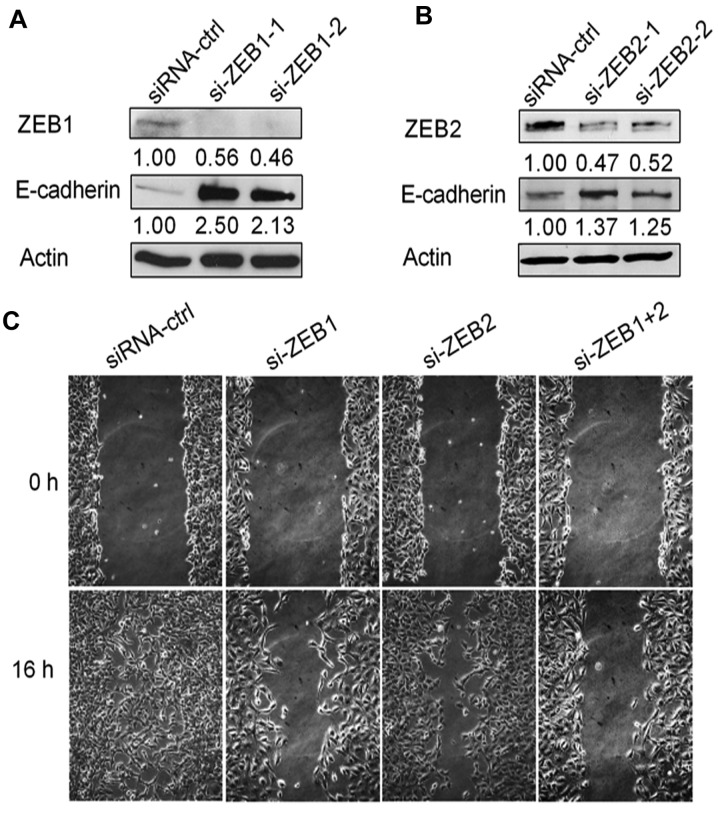
ZEB1 and ZEB2 are EMT enhancers. (A and B) Levels of E-cadherin protein in SKOV3 cells after transfection with si-ZEB1, si-ZEB2, or siRNA-ctrl were measured by western blotting using whole cell lysates from each sample. Actin was used to control protein loading. (C) Wound-healing assay in SKOV3 cells after transfection with si-ZEB1, si-ZEB2, or si-ZEB1+2; the extent of closure of the wound, representing cell migration, was monitored under phase-contrast microscopy and images were captured at 0 and 16 h. EMT, epithelial-to-mesenchymal transition.

To determine whether the inhibition of EMT by miR-101 was mediated by *ZEB1* or *ZEB2*, we knocked down *ZEB1* by two different siRNAs and found that both siRNAs led to increased E-cadherin protein levels in SKOV3 cells (Fig. 4A). Similarly, knockdown of *ZEB2* in SKOV3 cells by two different siRNAs led to the same result (Fig. 4B). We then performed the wound-healing assay in SKOV3 cells transfected with si-ZEB1 or si-ZEB2 or both. As expected, knockdown of ZEB1 or ZEB2 markedly decreased cell migration when compared with siRNA-ctrl, especially with dual silencing of both genes (Fig. 4C).

**Figure 5:**
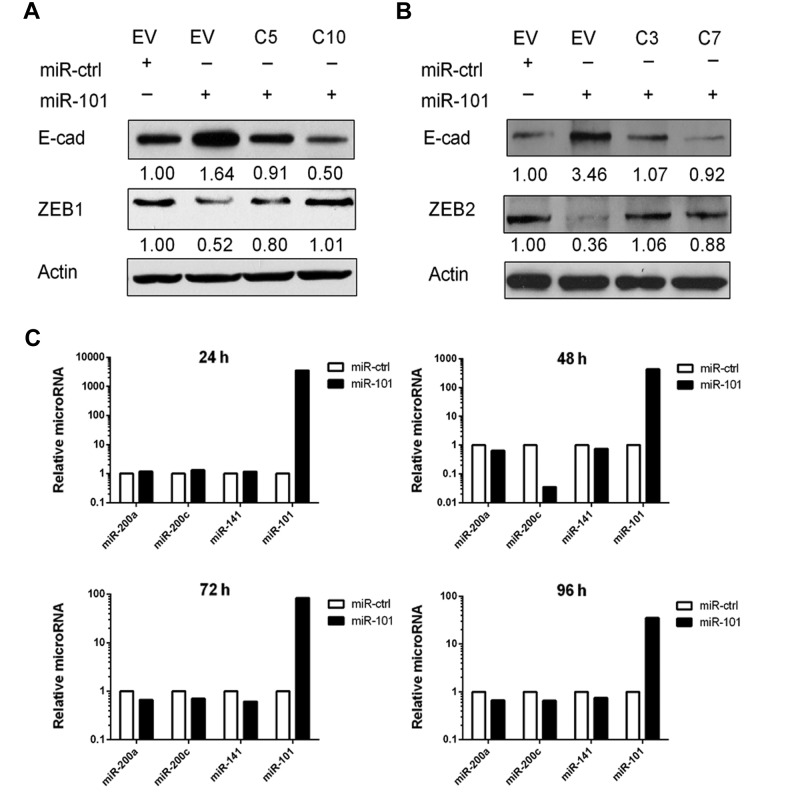
miR-101 suppresses EMT and increases E-cadherin expression independent of the miR-200 family. (A) Levels of E-cadherin (E-cad) and ZEB1 proteins in empty vector (EV) or ZEB1-overexpressing (C5 and C10, which are different clones) SKOV3 cells after transfection with miR-101 mimic (miR-101) or control (miR-Ctrl) were measured by western blotting using whole cell lysates from each sample. (B) Levels of E-cadherin and ZEB2 proteins in EV or ZEB2-overexpressing (C3 and C7, which are different clones) SKOV3 cells after transfection with miR-101 of miR-Ctrl were measured by western blotting using whole cell lysates from each sample. Actin was used to control protein loading. (C) Levels of miR-200a, miR-200c, and miR-141 in SKOV3 cells after miR-101 or miR-Ctrl transfection were analyzed by TaqMan miRNA quantitative PCR at 24, 48, 72 and 96 h. EMT, epithelial-to-mesenchymal transition.

We next performed ZEB1 and ZEB2 rescue experiments. To obtain stable clones, we first transfected SKOV3 cells with pcDNA3.1(+)-ZEB1 or pcDNA3.1(+)-ZEB2 that did not contain a 3′-UTR. When miR-101 was transfected into these cells, the expression of ZEB1 and ZEB2 was not downregulated and E-cadherin was not increased (Fig. 5A and B). These results showed that miR-101 upregulated E-cadherin through the miR-101-binding sites on the 3′-UTRs of *ZEB1* and *ZEB2*. We further examined the possibility that miR-101 may somehow increase expression of miR-200, which has been shown to also target the 3′-UTRs of *ZEB1* and *ZEB2*. We isolated RNA from miR-101- or miR-Ctrl-transfected SKOV3 cells and measured the level of miR-200 family members. We detected no significant changes in the levels of miR-200 family members in miR-101-transfected cells (Fig. 5C).

## Discussion

Ovarian cancer is the leading cause of mortality in gynecologic malignancies, as the high death rates from ovarian cancer remain largely unchanged over the past 30 years, with a 5-year overall survival rate of only 30–39% (26). A better understanding of the mechanisms involved in progression of ovarian carcinoma is urgently required. EMT has emerged as an important mechanism that promotes ovarian carcinoma progression. Through analysis of the Cancer Genome Atlas data, our previous study identified a master miRNA regulatory network that regulated EMT in serous ovarian carcinoma, and this network includes miR-101 (18). In the present study, we provided evidence for the first time that miR-101 suppresses EMT by directly targeting E-cadherin-suppressor genes *ZEB1* and *ZEB2* through specific binding sites on their 3′-UTRs.

There are two separate copies of the miR-101 gene, located on 1p31.3 and 9p24. Both regions have been identified as fragile regions of the genome that are associated with abnormal deletion or amplification in cancer (27). Downregulation of miR-101 has been observed in bladder cancer (28), intraductal papillary mucinous neoplasms of the pancreas (29) and ovarian carcinoma (30), suggesting that miR-101 plays a role in tumor suppression.

ZEB1 and ZEB2, members of the ZEB family, have been shown to induce EMT through repression of E-cadherin and to promote tumor progression and metastatic spread (31–33). Higher expression of ZEB1 and ZEB2 were found in diverse types of cancer (34–36) including ovarian carcinoma (37,38), suggesting that the two factors may play an essential role in EMT of ovarian carcinoma. The discovery that there are putative binding sites on the 3′-UTRs of both *ZEB1* and *ZEB2* genes for miR-101 suggests a potential mechanism through which miR-101 regulates EMT. Using reporter gene assays and rescue experiments, we validated this regulatory mechanism. Furthermore, ectopic miR-101 expression significantly upregulated E-cadherin and decreased mesenchymal markers and cell motility, indicating that miR-101 acted as a strong EMT suppressor in ovarian cancer cells.

Transcriptional regulators of E-cadherin expression include SNAI1, SNAI2, TWIST1, TWIST2, ZEB1 and ZEB2. These key regulators of E-cadherin expression can in turn be regulated by multiple miRNAs, revealing an impressive redundancy in regulation of this physiological process at multiple levels. Notably, both miR-200 (39) and miR-101 (30), both regulators of ZEB1 and ZEB2, are downregulated in ovarian carcinoma. Furthermore, miR-506, which regulates another E-cadherin repressor, SNAI2, is also downregulated in ovarian carcinoma (18). EMT in cancer is generally a late event associated with metastasis. This is consistent with the requirement that multiple redundant regulatory mechanisms be lost for EMT to occur.

With regard to the causes of the simultaneous loss of multiple miRNAs, results of our previous study, which showed that miR-506 expression is partially lost through methylation, suggested that deletion of miRNA genes may not explain this (18). Of note, recent reports showed that miR-200 family members as well as miR-101 are methylated in several types of cancer, explaining their decreased expression (40–42). It has been reported consistently that EZH2, which regulates the methylation program, may regulate E-cadherin expression in ovarian carcinoma (43). EZH2 has been proposed as an oncogene in many types of cancer including ovarian carcinoma (43,44). Conceivably, EZH2 upregulation may downregulate the EMT-suppressing miRNAs, removing a highly redundant mechanism in controlling E-cadherin expression. However, the redundant mechanism appears to be restricted to downregulation, as each of the suppressing miRNAs can be sufficient to inhibit EMT without requiring other miRNAs. This provides an opportunity for using one of these miRNAs as a therapeutic tool.

In conclusion, we demonstrated for the first time that miR-101 can directly target ZEB1 and ZEB2, resulting in suppression of the EMT in ovarian carcinoma. miR-101 may have therapeutic value in the treatment of ovarian carcinoma.
